# Recent Advances of Autophagy in Non-Small Cell Lung Cancer: From Basic Mechanisms to Clinical Application

**DOI:** 10.3389/fonc.2022.861959

**Published:** 2022-05-04

**Authors:** Weina Guo, Keye Du, Shanshan Luo, Desheng Hu

**Affiliations:** ^1^ Department of Integrated Traditional Chinese and Western Medicine, Union Hospital, Tongji Medical College, Huazhong University of Science and Technology, Wuhan, China; ^2^ Department of Neurosurgery, Union Hospital, Tongji Medical College, Huazhong University of Science and Technology, Wuhan, China; ^3^ Institute of Hematology, Union Hospital, Tongji Medical College, Huazhong University of Science and Technology, Wuhan, China; ^4^ Key Laboratory of Biological Targeted Therapy, The Ministry of Education, Wuhan, China; ^5^ Department of immunology, Hubei Clinical Research Center of Cancer Immunotherapy, Wuhan, China

**Keywords:** non-small cell lung cancer, immunotherapy, resistance, tyrosine kinase inhibitors, autophagy

## Abstract

Lung cancer is characterized by the most common oncological disease and leading cause of cancer death worldwide, of which a group of subtypes known as non-small cell lung cancer (NSCLC) accounts for approximately 85%. In the past few decades, important progression in the therapies of NSCLC has enhanced our understanding of the biology and progression mechanisms of tumor. The application of immunotherapy and small molecule tyrosine kinase inhibitors has brought significant clinical benefits in certain patients. However, early metastasis and the emergence of resistance to antitumor therapy have resulted in the relatively low overall cure and survival rates for NSCLC. Autophagy is a conserved process that allows cells to recycle unused or damaged organelles and cellular components. It has been reported to be related to the progression of NSCLC and resistance to targeted therapy and cytotoxic chemotherapy. Therefore, autophagy is considered as a potential therapeutic target for NSCLC. Mounting results have been reported about the combination of tyrosine kinase inhibitors and inhibitors of autophagy in models of NSCLC. This review aims to provide a comprehensive review on the roles of autophagy in NSCLC, focusing on related clinical data of agents that regulate autophagy in NSCLC. Furthermore, this study will provide a theoretical basis for further improvement of autophagy-based cancer therapy.

## 1 Introduction

Lung cancer including non-small cell lung cancer (NSCLC) and small-cell lung cancer (SCLC), has been reported to account for 11.6% and 18.4% of global cancer morbidity and mortality, respectively (1, 2). According to histological classification, approximately 85% of patients belong to the subtype referred to as NSCLC, among which the most common subtypes are lung squamous cell carcinoma (LUSC) and lung adenocarcinoma (LUAD) (3, 4). Over the past few decades, therapies for NSCLC have progressed from cytotoxic treatment to effective and better tolerated regimens that are designed to target to specific molecular subtypes (3, 5, 6). The identification of target gene alterations is an evolution for the lung cancer management, with the combination of tumor genotyping making personalized treatment possible, and it is of great benefit to patients treated with kinase inhibitors (TKIs) for EGFR, ALK, ROS1, BRAF, or MET (7–11). Furthermore, the introduction of immune checkpoint blockers (ICBs) such as monoclonal antibodies that target programmed death-1 (PD-1) or programmed death ligand-1 (PD-L1) and antibodies against cytotoxic T-lymphocyte antigen-4 (CTLA-4) have indicated a new direction for lung cancer care (12, 13). To further improve the treatment efficiency, there is an urgent need to deeply understand the mechanisms of acquired resistance so as to provide a theoretical basis for effective treatments at the time of emergence.

In 2016, Yoshinori Ohsumi was awarded the Nobel Prize in Medicine for his contributions in elucidating the genetic basis of autophagy (14, 15). autophagy is generally believed to be an evolutionarily conserved physiological process, which is triggered by cellular stress or nutrient depletion, leading to the circulation of intracellular compounds. The vesicle fuses with lysosomes, and through subsequent degradation, new metabolites are produced to meet cell metabolism and energy requirements (9, 16, 17). Actually, in mammalian cells, protein degradation during autophagy occurs through three different mechanisms, including macroautophagy, and two other relatively less studied types, namely microautophagy and chaperone-mediated autophagy (CMA) (18). In macroautophagy, double-membrane vesicles are formed through a closed restriction membrane, which separates cargo proteins from the rest of the cytoplasmic components. Interestingly, proteins enter the lysosome cavity through the invagination of the lysosomal membrane surface in microautophagy (18). Different from the above, the selective pool of cytosolic proteins degraded by CMA are directly translocated across the lysosomal membrane (19, 20).

As a matter of fact, autophagy has a Janus character in the initiation and progression of cancer. On the one hand, autophagy prevents carcinogenesis by reducing the damage of cells (including DNA), but once carcinogenesis occurs, the role of autophagy in energy balance would help in cultivating cancer cells, thereby helping these aggressive cancer cells to grow in the stress environment (21). This makes the role of autophagy not only limited to protecting the host, it also has a function that is not welcomed, including the promotion the recurrence and invasion of cancer. Results from genetically engineered mouse models (GEMMs) of lung cancer, pancreatic cancer and melanoma induced by mutations in RAS or BRAF indicated that autophagy inhibited the growth of early benign tumors, but accelerated the growth of advanced cancers (22–26). Furthermore, there is accumulating evidence indicating that autophagy inhibition could be a potential approach in the treatment of advanced cancer (27). Therefore, a more in-depth understanding of how autophagy affects events in cancer cells and the response of patients to treatment may contribute to the improvement of treatment regimen for NSCLC.

In this article, we will review the research progress on the role of autophagy in NSCLC progression, the mechanism of autophagy affecting NSCLC progression, as well as clinical results obtained so far using autophagy inhibitors in NSCLC. With the increasing results of clinical research focusing on autophagy, the review of these topics is particularly timely, which would enable us to target autophagy effectively to improve the clinical prognosis of patients with NSCLC.

## 2 The Process of Autophagy

**Figure 1:**
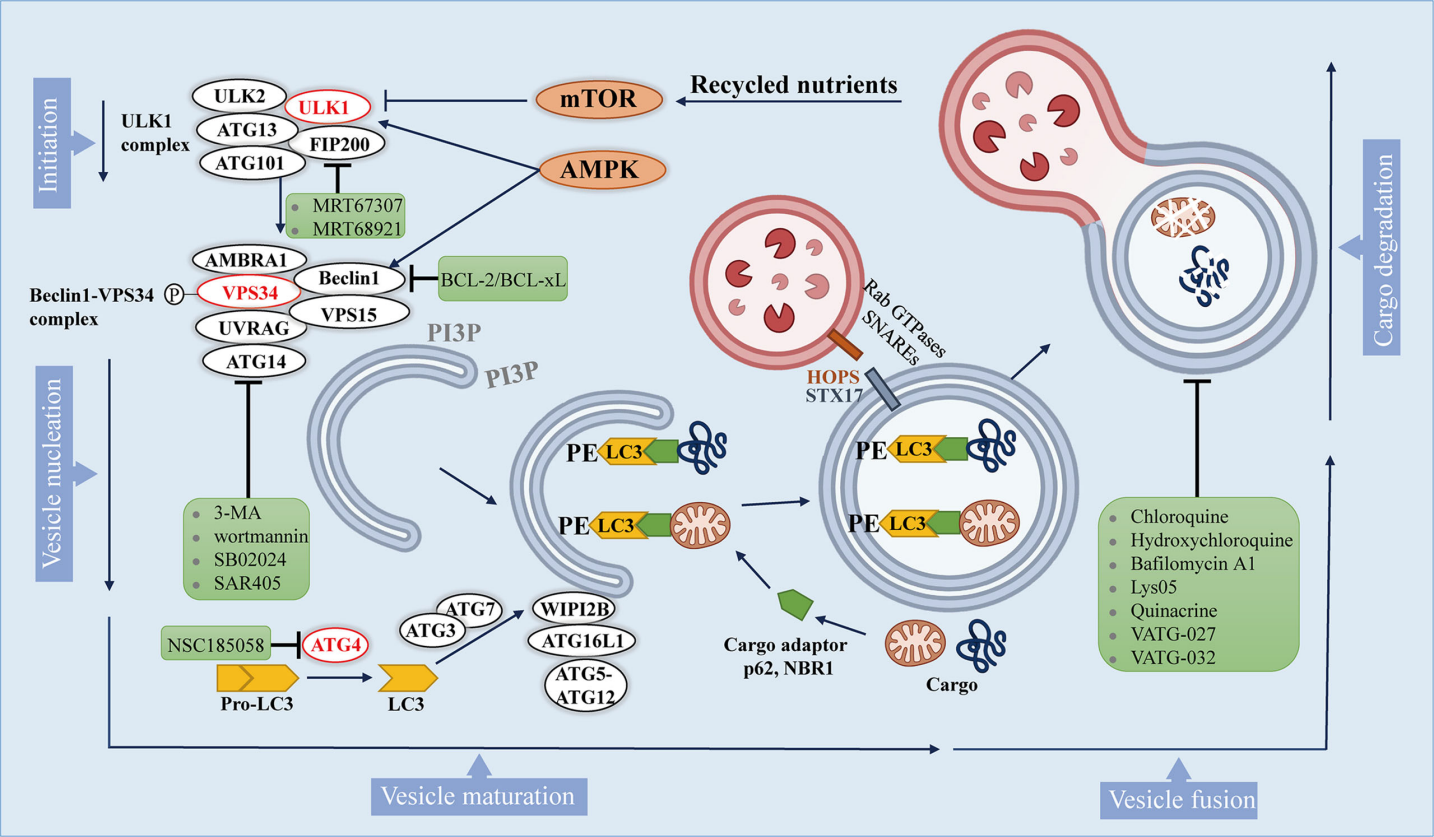
The autophagy pathway and multiple stages can be inhibited. The autophagy pathway consists 5 steps. Intracellular membranes are prepared by initiation and vesicle nucleation to form AVs through the formation of PI3P on membranes. Next, LC3-I is conjugated to PE on emerging AVs. Subsequently, LC3 is docked with the cargo adapter so that the cargo can be loaded into the AVs. After AVs matures, it fuses with lysosomes to complete the degradation of cargo and the recycling of nutrients. Autophagy inhibitors are shown in green boxes.

Macroautophagy (hereafter referred to autophagy) is a multistep lysosomal degradation pathway that supports nutrient recycling and metabolic adaptation, which has been implicated as a process that regulates cancer (14). Mastering the mechanisms of autophagy flux can promote the development of effective compounds, thus ultimately treating autophagy-related cancers. Based on our current knowledge, the autophagy pathway includes at least 5 steps, which are initiation, vesicle nucleation, vesicle maturation, vesicle fusion and cargo degradation (**Figure 1**).

Autophagy is initiated by activation of Unc-51-like kinase 1(ULK1) complex, which comprises ULK1, ULK2, autophagy-related gene 13 (ATG13), focal adhesion kinase interacting protein 200 kDa (FIP200) and ATG101. ULK1 complex can integrate two main stress signals in cells, including nutrient regulator (mTOR) and energy stress factor (AMPK). The ULK1 complex is usually inactive, and it is activated when mTORC1 is inhibited or AMPK is activated.

Once ULK1 kinase is activated, it would trigger the phosphorylation and activation of the Beclin1 -VPS34 (a class III phosphatidylinositol 3-kinase (PI3K)) complex, which includes Beclin1, VPS34, and other proteins such as activating molecule in BECN1-regulated autophagy protein 1 (AMBRA1), VPS15, ATG14, and UV radiation resistance associated gene protein (UVRAG), which depends on the subcellular localization of the complex (28). The activated Beclin1-VPS34 complex achieves vesicle nucleation through the formation of phosphatidylinositol 3-phosphate (PI3P) on membranes that can be derived from the endoplasmic reticulum (ER), mitochondria, plasma membrane (29–31).

During the maturation process, the formation of autophagosomes requires two unique protein conjugation events (32, 33): 1) ATG7 and ATG10 conjugate ATG5 to ATG12, and then ATG5-ATG12 binds to ATG16L1 to form a complex, and the ATG5-ATG12-ATG16L1 complex gets anchored onto PI3P produced by VPS34 on neonatal autophagosome through WIPI2B scaffold (34); 2) ATG4 cleaves pro-LC3 to generate soluble LC3-I, which is then conjugated to lipid phosphatidylethanolamine (PE) on the surface of the emerging autophagosome by ATG3 and ATG7, and further follows the guidance of the ATG5-ATG12-ATG16L1 complex (35). Once LC3-I is conjugated to lipid, it becomes inserted on the surface of the emerging autophagic vesicles (AVs) (36). On gel electrophoresis, the lipid-conjugated form of LC3 (LC3-II) migrates faster than LC3-I, so that the ratio of LC3-II and LC3-I can be used as an approximation of the number of AVs.

In addition to being a marker for AVs, LC3 on AVs is also a docking site for receptors of autophagy cargo, bringing autophagy cargo to AVs. Cargo receptors such as SQSTM1 (p62) and the neighbor of BRCA1 (NBR1) bind to proteins and organelles through ubiquitin labeling, and then undergo autophagy degradation (37). Specific cargo receptors will preferentially bind to specific cargoes, which may provide selectivity for the autophagy process (38). Once the isolation membrane is enclosed, it is called the autophagosome (27).

After autophagosomes are formed and cargos are sequestered, the cargo-bound autophagosomes are transported to the perinuclear region, where lysosomes exist (39). The membrane-tethering complexes (HOPS complex, VPS genes), Rab GTPases and soluble N-ethylmaleimide-sensitive factor attachment protein receptors (SNARE) along with syntaxin 17 (STX17) help the fusion of the autophagosomes to the lysosome (40, 41). Lastly, autophagic cargo are degraded by lysosomal hydrolases, and recycled contents are discharged through nutrient transporters, thereby fueling cell growth (42).

Although these 5 steps of autophagy are well established, additional autophagy regulators are still being discovered. These steps in the autophagy pathway represent potential drug targets, which provide pathways to influence autophagy positively and negatively.

## 3 The Role of Autophagy in NSCLC Progression

Evidence suggests that the role of autophagy in tumorigenesis may be dichotomous. On the one hand, mice with allelic loss of Beclin1 are tumor prone and liver-specific deletion of ATG5 or ATG7 induces benign hepatomas, which suggest a role for autophagy in tumor suppression (43–45). On the other hand, autophagy enables cancer cells to survive from metabolically stressed and hypoxic regions in solid tumors (46–48).

Interestingly, Rao et al. (49) found that the inactivation of the essential autophagy gene ATG5 at early stage increased the number and volume of hyperplastic regions and adenomas in the mouse model of KRAS-driven NSCLC. Conversely, at later stages, autophagy is required for the progression of adenomas to adenocarcinomas. Indeed, the role of autophagy in cancer is environment-dependent, and its upregulation is necessary for cancer cells to survive in hypoxic tumor regions (50). Moreover, the transformation of the oncogene RAS up-regulates the basal level of autophagy to meet the needs of maintaining mitochondrial metabolism and tumor progression (23, 51, 52). Data from several studies revealed that, in KRAS^G12D^-and BrafV600E-NSCLC in adult mice, loss of ATG7 caused tumors to accumulate defective mitochondria and leaded to impaired metabolism. On the other hand, in the absence of ATG7, cancer cell proliferation is inhibited, and the tumor develops into benign eosinophil tumor instead of adenoma and cancer, thereby prolonging the lifespan of mice (53–55). Karsli-Uzunbas (56) et al. found that, in the models of NSCLC, 5 weeks of acute reduction in autophagy transformed lung adenocarcinoma into oncocytomas, and blocked the signal transduction of mTOR and MAP kinase, as well as cell proliferation and survival.

Tumors that have formed are more dependent on autophagy than newly developed tumors and normal tissues. This indicates that there may be a therapeutic target to inhibit tumorigenesis by appropriately controlling the extent and timing of autophagy inhibition, while preserving most of the normal tissues. Therefore, a comprehensive understanding of tumor dependence on the autophagy pathway driven by specific oncogenic events can promote autophagy regulation as an effective and specific cancer treatment strategy.

## 4 The Mechanism of Autophagy Affecting NSCLC Progression

### 4.1 Autophagy Shapes the Tumor Microenvironment of NSCLC

**Figure 2:**
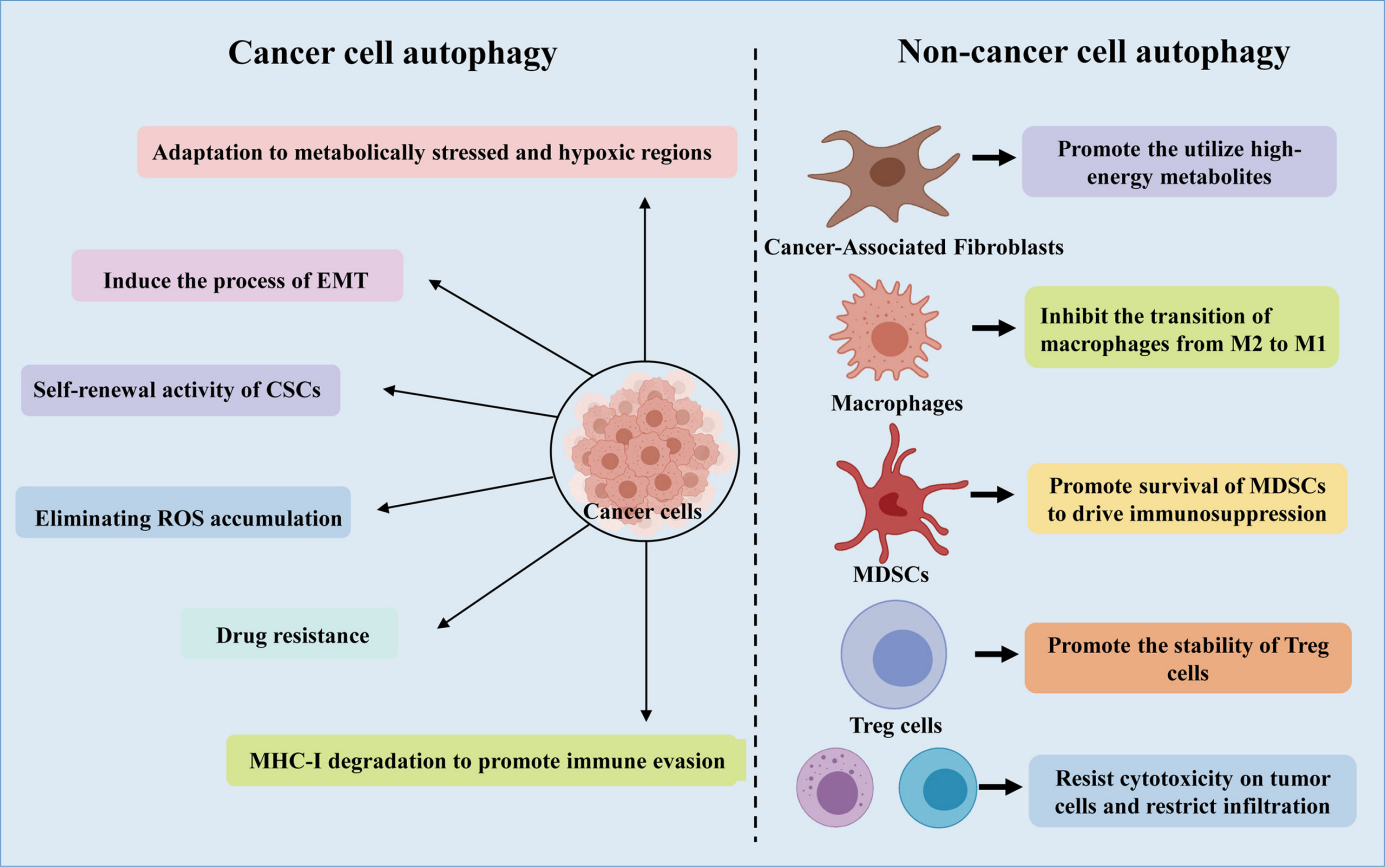
A schematic representation of the role of autophagy in cancer cells and non-cancer cells.

The tumor microenvironment (TME) is shaped by several processes, such as autophagy and immune responses (57). The TME takes advantage of autophagy to meet the metabolic needs of cancer stem cells (CSCs), sounding immune cells, cancer associated fibroblasts (CAF), angiogenesis, neural connections, as well as extracellular matrix (**Figure 2**) (21). Furthermore, recent studies have shown that there is a complex interaction between autophagy and epithelial-mesenchymal transition (EMT), through which cancer cells acquire invasive phenotype and metastatic potential (58, 59).

#### 4.1.1 CSCs

CSCs are a subgroup of cancer cells, which can promote the occurrence and development of cancer and are related to the production of drug resistance. Interestingly, it is reported that CSCs promote and maintain tumor heterogeneity by activating EMT, Juxtacrine and inflammatory signals in TME (60). It has been revealed that Lung CSCs can degrade p53 through the autophagy pathway, thereby enhancing Zeb1 expression and regulating stemness, suggesting that the autophagy-p53-Zeb1 axis regulates the self-renewal ability of CSCs (61). Moreover, in lung cancer stem cells, miR-138-5p mimic can inhibit ATG7-dependent regulation of autophagy and self-renewal (62).

#### 4.1.2 CAFs

NSCLC has a high stromal content, which contribute to low response rates to current therapies and a poor long-term survival (63). As one of the most abundant cell types in the tumor stroma, CAFs have a tremendous influence on remodeling the stromal compartment within the TME through collagen deposition and matrix metalloproteinase secretion (64). *In vitro* and *in vivo* analysis using xenograft models of lung cancer indicated that CAFs produced IGF1/2, CXCL12 and β-hydroxybutyrate and increased the level of reactive oxygen species (ROS), which resulted in mTOR inactivation and autophagy increasement in cancer cells after irradiation (65). In addition, by triggering ROS-mediated autophagy in neighboring CAFs, cancer cells can use high-energy metabolites like glutamine and lactic acid to carry out the tricarboxylic acid (TCA) cycle under stress conditions, thereby supporting tumor growth and progression (21).

#### 4.1.3 EMT

Recently, autophagy has been connected to EMT, an indispensable multistep process required for cancer cells’ invasion and metastasis (66, 67). Moreover, studies have also shown that EMT induced by transforming growth factor (TGF)-β1 in NSCLC is autophagy-dependent (68). In addition, rapamycin-induced autophagy can activate cell migration, invasion and the expression of EMT markers, and knockdown of Beclin1 can reverse this phenomenon (69). Another hepatocellular carcinoma model showed that inhibiting autophagy *in vitro* did not alter migration, invasion and EMT marker expression, while inhibition of autophagy *in vivo* caused cells to be sensitive to anoikis and reduced lung metastases (70).

There still exists many questions for cancer and TME treatment targeting autophagy. To unravel the signal transduction that controls the interaction between cancer cells and other components of TME, one of the future focuses may be to develop new models. For example, the development of 3D co-cultivation system might reveal some important metabolic interactomes in the TME. In order to have a better future for autophagy targeted cancer therapy, further basic research and translation studies are needed to clarify new findings and solve unanswered questions, like the role of bacterial components in the tumor microbiome.

### 4.2 Autophagy and Metabolic Reprogramming in NSCLC

Some studies have proved the importance of autophagy in maintaining the growth and survival of cancer cells by regulating the metabolism of cancer cells. Interestingly, autophagy enhances glucose uptake by up-regulating the expression of glucose transporter type 1 (GLUT1) on the cell surface, while blocking autophagy leads to accumulation of GLUT1 in late endosomes (71). Moreover, it is reported that during glucose starvation, hexokinase-2 (HK2) converts cell metabolism to autophagy-dependent pathway from glycolysis-dependent ones through the inhibition of mTORC1 (72). Deregulation of HK2 in Tongue Squamous Cell Carcinoma inhibited autophagic activity and weakened the invasiveness (73). Furthermore, cystine transporter SLC7A11-mediated cystine introduction depends on autophagy-mediated localization on the cell, and inhibition of autophagy would result in inactivation of SLC7A11 (74).

As an important event involved in the processing of metabolites and biosynthesis, autophagy can promote the metabolic adaptation of cancer cells in the survival of TME (**Figure 2**). Guo et al. found that glutamine or glutamate can rescue the starving ATG7-deficient KRAS-driven lung cancer cells, revealing the important role of autophagy in supporting the cyclic metabolites of TCA and nucleotide synthesis (25). In addition, adult mice with acute systemic loss of ATG7 died during fasting. The mice showed obvious muscle atrophy and died of hypoglycemia, which indicated that autophagy is necessary to maintain glucose homeostasis (56). Interestingly, the survival rate of cancer cell lines lacking ATG7 and p53 is reduced, and lipid cysts are formed, showing dysfunction of lipid metabolism (55). Additionally, in patients with NSCLC expressing a mutant form of EGFR, c-Jun n-terminal kinase (JNK)-induced autophagy results in high levels of glycolysis. Based on these phenomena, inhibition of autophagy may be a potential therapy for the treatment of lung adenocarcinoma.

### 4.3 Autophagy and ROS

ROS participates in the occurrence and development of cancer by oxidizing cell lipids, damaging the integrity of DNA and proteins, which also makes them more susceptible to the aggression of cancer (21). Autophagy has been shown to closely interplay with ROS (75–77). In the process of tumorigenesis, the production of ROS is related to the accumulation of dysfunctional organelles, which activates the autophagy pathway to clear the damaged organelles in the cells. In turn, the loss of autophagy can further induce ROS formation, leading to DNA damage (78). Autophagy eliminates accumulated ROS and relieves the metabolic stress of cancer cells in the TME, thereby promoting tumor survival (23, 42) (**Figure 2**). Cancer cells produce ROS under hypoxic conditions, and the transfer of ROS to CAFs promotes autophagy, thereby providing nutrition for the growth of cancer cells (21). Significant increase in ROS levels can cause DNA damage and the transformation of metabolism from OXPHOS to glycolysis, proving that autophagy could promote cancer cell growth by controlling ROS levels and energy metabolism (23). However, the role of ROS in the regulation of the progression of NSCLC by autophagy remains to be further determined.

## 5 Clinical Relevance of Autophagy in NSCLC

### 5.1 Autophagy and Drug Resistance in NSCLC

It is worth noting that more and more studies have shown that autophagy is closely related to drug resistance in NSCLC. For a long time, the emergence of resistance to EGFR inhibitors has been a crucial clinical issue (79). Erlotinib can induce apoptosis and autophagy in NSCLC cells with EGFR activating mutations, and inhibiting the autophagy process can enhance the cytotoxicity of erlotinib to cancer cells (80). In addition, by inhibiting autophagy in NSCLC cells with wild-type EGFR, the resistance of NSCLC cells to erlotinib can be eliminated (81). Moreover, the inhibition of autophagy in TKI-resistant lung cancer cells can significantly enhance the sensitivity of lung cancer cells to erlotinib by regulating the endoplasmic reticulum stress (82). Coincidentally, Han W and other studies have shown that EGFR-TKIs, such as gefitinib and erlotinib, can activate autophagy of human lung cancer cells, and then the growth inhibitory effect of EGFR-TKIs on cancer cells is weakened (83). *In vitro* study using cell lines and clinical samples showed that one of the mechanisms of EGFR-TKI resistance is LC3a-mediated autophagy activation (79). Furthermore, other pre-clinical studies have demonstrated that the inhibition of autophagy can overcome the emergence of resistance to tyrosine kinase inhibitors in NSCLC and ALK-positive lung cancer (84, 85). Beyond that, it has been proven that hypoxia-induced autophagy in lung cancer leads to resistance to the chemotherapy drug cisplatin (86). Based on the phenomenon that EGFR-TKIs induce autophagy (83, 87), and autophagy may lead to chemotherapy resistance (88), researchers speculate that autophagy may be a protective mechanism for cancer cells and contribute to the emergence of drug resistance in NSCLC.

Elucidating the role of autophagy in drug resistance will aid in exploring how to manipulate autophagy to maximize the effect of cancer therapy. As the link between autophagy and drug resistance continues to strengthen, autophagy will undoubtedly become a promising target in cancer therapy. At the same time, it is also urgent to advance the combination therapy of autophagy modulators and existing antitumor drugs in clinical trials.

### 5.2 Autophagy Promotes Tumor Evasion in Antitumor Immune Responses

Autophagy has been reported to modulate immune components, mainly containing T and B lymphocytes, natural killer (NK) cells, tumor-associated macrophage (TAMs), and dendritic cells (DCs), Myeloid-derived suppressor cells (MDSCs), thereby interfering with host innate and adaptive immune responses. Autophagy in immune cells located in TME controls host antitumor immunity and induces an immunosuppressive microenvironment **(** **Figure 2**).

**Figure 3:**
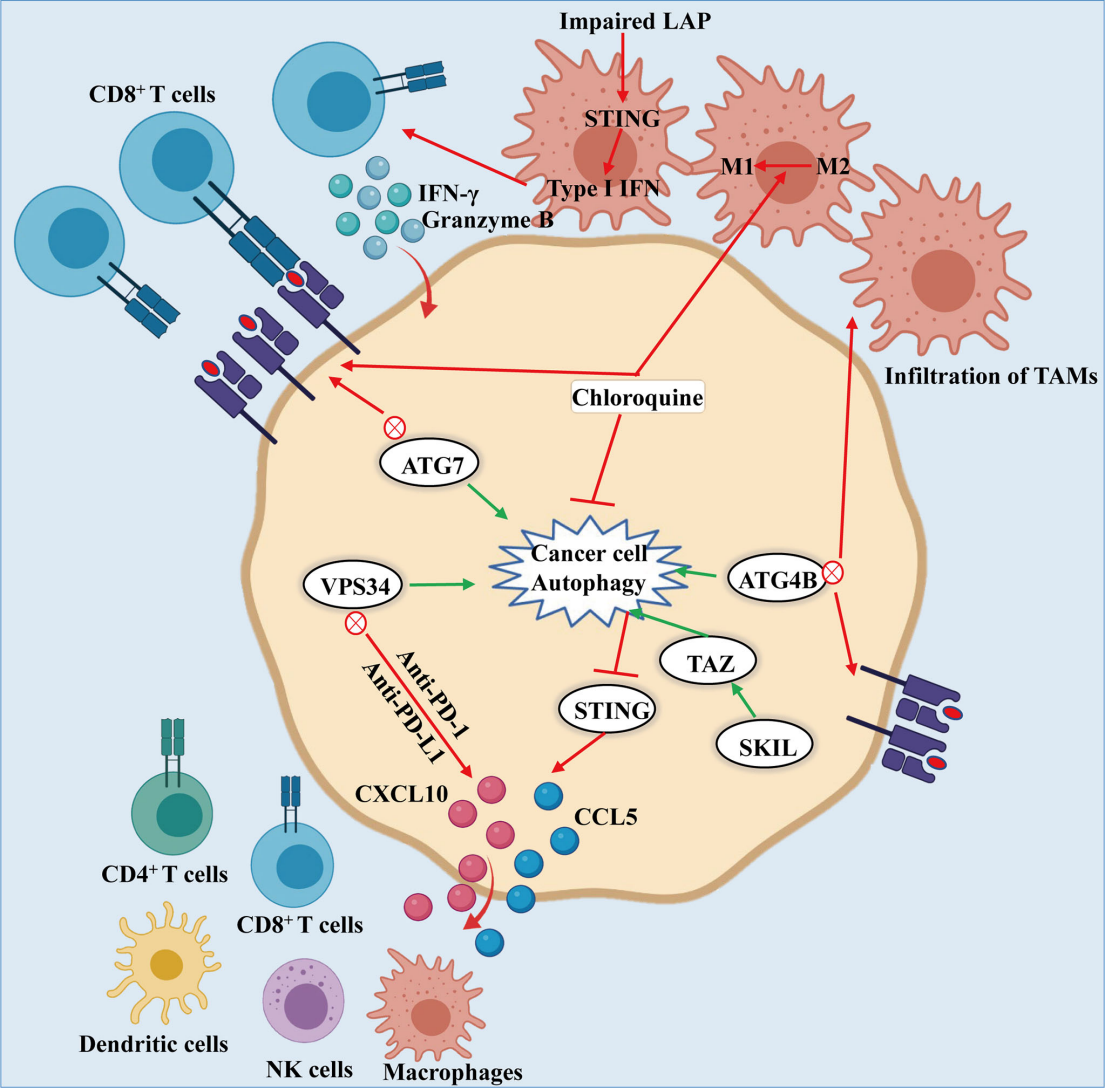
Autophagy-mediated immune evasion of cancer cells: Knockdown of ATG7, or dominant negative expression of ATG4B or treatment of chloroquine leads to the inhibition of autophagy, which induces the accumulation of MHC-I on the surface of cancer cell. The MHC-I accumulation promotes the recognition and effect of CD8^+^ T cells on cancer cells. Likewise, inhibiting autophagy results in the infiltration of TAMs and the conversion of macrophages from M2 to M1 phenotype, thereby enhancing the antitumor activity. What is noteworthy is that impairment of LAP results in activation of T cells mediated by STING, producing granzyme B and IFN-γ to kill the cancer cells. In addition, the combination of PIK3C3/VPS34 inhibitors with anti-PD-1 and PD-L1 therapy could increase the numbers of NK and CD8^+^, CD4^+^ T cells, macrophages and dendritic cells along with CCL5 and CXCL10 infiltrating in tumor environment. Moreover, SKIL promoted tumorigenesis and immune escape of NSCLC cells by up-regulating the TAZ/autophagy axis and inhibition on downstream STING pathway, thereby resulting in reducing T cell infiltration and release of chemokines including CXCL10, CCL5 and IFN-β.

Upon systemic autophagy inhibition by chloroquine (CQ), as well as tumor-specific autophagy inhibition, infiltration of CD8^+^ T cells and an increase of MHC-I molecules on the surface of cancer cell make them sensitive towards ICB, thereby inhibiting the growth of tumors (89, 90). The combination of anti-PD-1/PD-L1 blockade and Vps34 inhibition promotes the mass production of pro-inflammatory cytokines and chemokines CCL5, CXCL10 and IFN-γ, as well as the accumulation of CD4^+^, CD8^+^ T and NK cells, DCs and M1 macrophages, thus enhancing the efficacy of treatment (**Figure 3**) (91). In addition, autophagy can inhibit the antitumor immune responses by triggering the degradation of cytotoxic granules released from CD8^+^ T and NK cells (92, 93). Furthermore, the combination of antitumor drug 5-FU and CQ can augment the response of CD8^+^ T cells to HCT-116 colon cancer cells and promote the maturation of DCs (94). It is worth noting that the conditional deletion of ATG7 in KRAS^G12D^-driven lung cancers closely correlated with abundant tumor infiltration by CTLs and macrophages (55, 95). Interestingly, Ma et al. found that SKIL promoted tumorigenesis and immune escape of NSCLC cells through upregulation of TAZ/autophagy axis and inhibition of downstream STING pathway, resulting in decreased T cell infiltration and release of chemokines such as CXCL10, CCL5 and IFN-β (96).

Besides that, specific deletion of two essential genes, ATG7 or ATG5, in Treg cells impaired their survival fitness and lineage stability, leading to loss of Treg and greater tumor resistance (97). In addition, it was shown that by inducing autophagy to promote the survival of MDSCs, the high mobility group box 1 can induce an immunosuppressive tumor microenvironment, thereby promoting tumor progression (98). Furthermore, glycolysis inhibits the formation of autophagy, and enhances the expression of autophagy-mediated partial hepatic enrichment activating factors, thereby promoting the expression of granulocyte-macrophage colony stimulating factor, which supports the development of MDSCs in tumors (99).

In most solid tumors, autophagy plays a crucial role in controlling macrophages at different stages, especially the polarization. Interestingly, in B16 melanoma and H22 liver cancer tumor-bearing mouse models, CQ treatment promotes the antitumor immunity mediated by CD8^+^ T cells *via* activating the inflammatory cytokines, thereby causing TAMs to deviate from conversion of M2 phenotype to M1 phenotype (100). Interestingly, LC3-associated phagocytosis (LAP) has been shown to contribute to the polarization of macrophages towards M2 phenotype in TME (101). Larissa D et al. found that, upon phagocytosis of dying cancer cells, LAP-deficient TAMs induce antitumor T cell responses by triggering STING-mediated type-I interferon responses and augmenting the expression of pro-inflammatory gene (101).

It is noteworthy that inactivation of the autophagy gene ATG5 leads to accelerated tumorigenesis at early stages by promoting the infiltration of Treg cells in a mouse model of NSCLC (49). Therefore, great importance should be attached to the right staging and grading of tumors to maximize the efficacy of autophagy inhibitors from the perspective of clinical application.

### 5.3 Biomarkers of Autophagy in NSCLC

A major challenge in all of the clinical studies has been identifying appropriate pharmacodynamic biomarkers which are specific in evaluating changes within autophagy. Nevertheless, there are few effective and specific autophagy-related biomarkers currently identified, which are crucial for selecting patients for autophagy inhibitor-related clinical trials and evaluating the effect of treatment. See **Table 1** for some examples of autophagy-related proteins with biomarker potential.

**Table 1:** Autophagy-related proteins as biomarkers.

Biomarker	Setting	Study object	Clinical significance	Reference
LC3B	Colorectal cancers (CRC)	127 CRC patients with known KRAS mutational status	LC3 overexpression was significantly associated with decreased overall survival (OS) in the KRAS-mutated CRC subgroup	(102)
	Breast cancer	20 breast cancer patients	Elevated expression of LC3B was associated with increased nuclear grade and shortened survival.	(103)
	Hepatocellular carcinoma (HCC)	156 operable HCC patients	Overexpression of LC3B correlates with malignant progression and predicts a poor prognosis in HCC	(104)
	non-small cell lung cancer (NSCLC)	466 stage I/II NSCLC patients	High LC3B levels may be correlated with lower tumor aggressiveness	(105)
LC3A	breast carcinomas	102 operable breast carcinomas patients	“Stone-like” structure (SLS) distribution of LC3A was related to high-grade tumors and a less favorable outcome	(106)
	NSCLC	115 patients with NSCLC treated with surgery	Elevated presence of SLSs is strongly linked to a poor outcome in NSCLC	(107)
p62	NSCLC	466 stage I/II NSCLC patients	High expression of p62 was significantly associated with higher tumor aggressiveness	(105)
		104 NSCLC patients	High expression of p62 was significantly associated with shorter survival	(108)
		109 NSCLC patients	The accumulation of p62 was associated with worse lung cancer‐specific survival	(109)
ULK1/2	HCC	156 operable HCC patients	ULK1 expression was negatively correlated with 5-year progression free survival	(110)
Beclin1	NSCLC	104 NSCLC patients	Low expression of Beclin1 was significantly associated with shorter survival	(108)
		244 primary NSCLC patients	Low expression of Beclin1 showed significantly inferior OS and progression-free survival	(111)
		1159 patients with NSCLC	High level of Beclin1 was significantly associated with better OS in NSCLC	(112)
ATG4B	Oral squamous cell carcinoma (OSCC)	498 OSCC patients	High protein levels of ATG4B were significantly associated with worse disease-specific survival	(113)
LAMP2A and HSC70	Pulmonary squamous cell carcinomas (pSQCC)	402 patients with primary resected pSQCC	High LAMP2A and HSC70 expression were associated with worse OS and disease-free survival	(114)
Autophagy-related lncRNAs	Lung adenocarcinoma (LUAD)	Data from The Cancer Genome Atlas database	16 autophagy-related lncRNAs were identified to have significant prognostic value for LUAD patients	(115)

#### 5.3.1 LC3B

Microtubule-associated protein 1 light chain 3B (MAP1LC3B, LC3B) is one of the best studied proteins in autophagy-related proteins, and has been utilized as an autophagy marker in multiple trials *in vivo* and *in vitro*. Accumulating evidence showed that the high expression of LC3B is related to the high aggressiveness and adverse prognosis of many types of cancers, including colorectal cancers (102), breast cancer (103) and hepatocellular carcinoma (104). It’s worth noting that a recent study of NSCLC evaluated the relationship between the expression levels of LC3B and p62 and prognosis, and found that high punctate expression of LC3B may be associated with a good prognosis (105).

#### 5.3.2 LC3A

Early studies reported that there are three different distribution patterns of LC3A in solid tumors through immunohistochemical staining, including diffuse distribution in the cytoplasm, paranuclear and “stone-like” structure (SLS) distribution (106), and each distribution pattern represents a different prognostic result. It is worth noting that the increase in the number of SLS is related to the adverse prognosis of NSCLC (107).

#### 5.3.3 p62

In the process of autophagosome formation, p62 acts as a bridge linking LC3 and its substrates (116). Since p62 is degraded in the autophagy flux, it is generally believed that the accumulation of p62 protein in the cell is a sign of the inhibition of autophagy (19). In NSCLC, the high expression of p62 significantly related to the tumor’s high aggressiveness and poor prognosis (108, 109).

#### 5.3.4 ULK-1/2

ULK-1 and ULK-2 are the only serine/threonine kinases in the process of autophagy (117, 118), and small molecule inhibitors for ULK-1/2 are under development (118, 119). In hepatocellular carcinoma, ULK1 expression was reported to be negatively correlated with 5-year progression free survival (110). However, no association of ULK-1/2 with prognosis in NSCLC is available from current researches. To better determine the prognostic value of ULK1 and ULK2 in different cancer types, more studies are urgently needed in larger patient cohorts.

#### 5.3.5 Beclin1 and VPS34

As a key regulator of autophagy, Beclin1 was reported to be an independent prognostic biomarker in the NSCLC (111). Similarly, Zheng et al. (112) reported that the high expression of Beclin1 is related to the better prognosis of NSCLC, indicating that Beclin1 may become a favorable prognostic marker of NSCLC.

#### 5.3.6 ATG4B

As the enzymatic roles of cysteine protease ATG4B are of great significance in the process of autophagy, it is currently referred as one of the potential therapeutic targets (120, 121). Intense presence of ATG4B was significantly associated with worse disease-specific survival in oral squamous cell carcinoma (113). Previous researches revealed increased expression of ATG4B in lung cancer cells (122), but its prognostic value for different cancers is poorly understood.

#### 5.3.7 Additional Autophagy-Related Biomarkers

Of course, there are also reports on the potential of other autophagy-related proteins as biomarkers. For instance, in primary resected squamous cell carcinomas of the lung, chaperone-mediated autophagy markers LAMP2A and HSC70 have been identified as independent poor prognostic markers (114). In addition, Jiang et al. (115) identified 16 autophagy-related long non-coding RNAs (lncRNAs) which have significant prognostic value for LUAD patients.

### 5.4 Autophagy Can be Inhibited at Multiple Stages

Formation of the autophagosome requires the assistance of various genes called autophagy related (ATG) genes, which are evolutionarily-conserved (123). Interestingly, accumulating evidence revealed that autophagy can be inhibited at multiple stages (**Figure 1**). It’s reported that two drugs MRT67307 and MRT68921 have been synthesized to inhibit ULK1 and ULK2 specifically, which lead to the inhibition of autophagy flux (119).

After the phosphorylation of Beclin1 by ULK1, it promotes the localization of autophagic proteins to the phagophore. It is being proved that the pro-autophagic activity of Beclin1 can be attenuated since BCL-2 and BCL-xL can interact with Beclin1 at the BH3 domain (124). In addition, phosphorylation of VPS34 (also known as PIK3C3) reduces its interaction with Beclin1, which can be targeted pharmacologically within the upstream by 3-methyladenine (3-MA) and wortmannin (125) which can inhibit PI3K, or VPS34 inhibitors, like RNAi or SB02024, SAR405 (91, 126).

The process of growing double membranes undergoing maturation and finally forming autophagosomes requires the participation of a variety of enzymes, including ATG4B, ATG7, and ATG10. Studies have shown that the ATG4B inhibitor NSC185058 (121) have both *in vitro* and *in vivo* antitumor activity. Besides, another study found that inhibition of autophagy by knocking down ATG7 or expressing dominant negative ATG4B in cancer cells resulted in a significant increase in the number of CD8^+^ T cells infiltrating in pancreatic tumors (89).

Later on, protein STX17 promotes the fusion of autophagosomes and lysosomes to produce autolysosomes. In this regard, CQ or hydroxychloroquine (HCQ) and bafilomycin A1 can inhibit the fusion of autophagosomes with lysosomes, and lysosomal inhibitors such as Lys05, quinacrine, VATG-027 and VATG-032 can also be utilized to target this process to inhibit autophagy (127–131).

As an autophagy inhibitor, the clinical efficacy of CQ undoubtedly illustrates the promise of autophagy inhibition as a therapeutic strategy, but also highlights the critical need for new inhibitors of autophagy, including the development of new compounds, as well as promotion of translation into clinical medicine. In this regard, certain inhibitors mentioned above are showing initial promise.

### 5.5 Clinical Trials Targeting Autophagy in NSCLC

Indeed, clinical intervention trials targeting autophagy in cancer treatment are already underway, most of which focus on inhibition of autophagy. In March 2022, a search for the search term “autophagy and cancer” on the ClinicalTrials.gov website showed 91 studies, mainly focusing on inhibiting and evaluating autophagy to improve prognosis of cancer patients.

As the only drugs used to inhibit autophagy in current clinical practice, CQ and hydroxychloroquine (HCQ) can prevent the degradation of cargo by deacidifying the lysosome and blocking the fusion of autophagosomes with lysosomes (132). Early clinical evidence for improving treatment effects by inhibiting autophagy comes from a small trial involving 18 patients with glioblastoma, which revealed that the median survival of patients receiving CQ combined with radiotherapy and temozolomide alkylation treatment was significantly prolonged compared with the control group (133). Hereafter, a randomized, double-blind, placebo-controlled trial demonstrated that administration of CQ in addition to the conventional treatment of glioblastoma multiforme can improve the mid-term survival rate (134).

The integrated results of published clinical trials (**Table 2**) indicate that HCQ is safe for the treatment of NSCLC. Interestingly, a dose-escalation phase I study was conducted with erlotinib and HCQ in patients with advanced NSCLC who had previously temporarily benefited from EGFR inhibitor therapy. They found that taking HCQ 1000 mg daily was tolerable and safe for patients. One patient had a partial response to the combination of erlotinib and HCQ, and the overall response rate was 5% (135). Furthermore, seventy-three patients with NSCLC or breast cancer with brain metastasis were randomly grouped, with patients receiving full brain radiotherapy and 150 mg of CQ per day for 4 weeks or the same schedule of full brain radiotherapy and a matching placebo. In their study, they found that combination of full brain radiotherapy and CQ improved the control of brain metastasis with no increase in toxicity (136). In addition, a phase Ib/II report of chemotherapy with HCQ revealed that addition of HCQ could reverse chemotherapy resistance in advanced NSCLC (137).

**Table 2:** The ongoing clinical trials using therapy targeting autophagy in NSCLC.

Clinical trial identifier	Autophagy Inhibitor	Clinical trial Phase	Additional treatment
NCT01649947	Hydroxychloroquine	II	Paclitaxel Carboplatin Bevacizumab
NCT00728845	Hydroxychloroquine	II	Paclitaxel Carboplatin Bevacizumab

Clinical trials, in which CQ or HCQ were utilized as autophagy inhibitors that targeting lysosomes, have proved the safety of targeting autophagy in the treatment of cancer. In addition to lysosomal inhibitors, other autophagy-specific inhibitors are still under development, including drugs targeting early steps, such as ATG4B and ULK1. Although preliminary data are encouraging, these compounds are still in early preclinical studies (138).

## 6 Conclusion and Future Perspective

As the leading cause of cancer-related death, NSCLC remains difficult to cure. Elucidating the molecular mechanisms of NSCLC and discovering new biomarkers will aid in developing more specific and efficient therapies. Autophagy is an important physiological activity that controls cell survival and death, affecting cell homeostasis and clinical therapeutics. With an increasing understanding of the role and mechanisms of autophagy, the problems we currently face are clearly more complex than initially anticipated. Above all, we need to identify some reliable compounds targeting key components of autophagy to deepen our understanding of the consequences of pharmacological regulation of autophagy and help translate it into clinical use. In clinical trials, investigating the clinical efficacy of currently available autophagy-modulating compounds will improve our understanding of the effects of these autophagy-modulating agents and promote its translation in clinical applications. In clinical trials, studying the clinical efficacy of currently available autophagy-modulating compounds helps to deepen our understanding of the effects of these autophagy-modulating agents, thus establishing relevance to preclinical models.

Autophagy is essential for maintaining glucose homeostasis and tumor growth in lung cancer (139). Based on this fact, it’s reasonable to assume that tumor growth can be restricted through the inhibition of autophagy. Notably, there is also evidence that autophagy deficiency triggers inhibition of antitumor immunosurveillance (49), which undoubtedly points to other therapeutic concepts. Overall, the fact that autophagy has been described as both tumor suppressor and tumor promoter in NSCLC does not mean that it cannot be therapeutically modulated. The overwhelming evidence points to inhibition of autophagy in NSCLC, and the results of the combination of chemotherapeutics and autophagy inhibition have led to the initiation of several clinical trials of chemoradiotherapy in combination with hydroxychloroquine (NCT01649947; NCT00728845). The predictive value of mouse models is limited due to the significant differences in immune system function between mice and humans, as well as the inherent limitations of oncogenic induction and genetically engineered models. Therefore, the impact of modulating autophagy on antitumor immune responses still needs to be evaluated in clinical trials.

There is still no complete and reliable system to assess autophagy in human samples, including blood and tumors, which undoubtedly limits our ability to evaluate autophagy regulation in clinical trials. In order to have a better future for autophagy targeted cancer therapy, further studies should focus on developing better biomarkers as pharmacodynamic markers for the efficacy of autophagy inhibitors and patient selection in the treatment. At the same time, it is obvious that, as tool compounds and autophagy inhibitors, stronger and specific autophagy inhibitors are needed.

## Author Contributions

Conceptualization, WG and KD. Investigation, WG and KD. Writing—original draft preparation, WG and KD. Writing—review and editing, WG, KD, SL, and DH. Project administration, SL and DH. All authors have read and agreed to the published version of the manuscript.

## Funding

This work was funded by the National Key R&D Program of China (no. 2019YFC1316204), the National Natural Science Foundation of China (82161138003, 81974249, 82070136), the Natural Science Foundation of Hubei Province (2020BHB016).

## Conflict of Interest

The authors declare that the research was conducted in the absence of any commercial or financial relationships that could be construed as a potential conflict of interest.

## Publisher’s Note

All claims expressed in this article are solely those of the authors and do not necessarily represent those of their affiliated organizations, or those of the publisher, the editors and the reviewers. Any product that may be evaluated in this article, or claim that may be made by its manufacturer, is not guaranteed or endorsed by the publisher.

## References

[1] Imyanitov EN Iyevleva AG Levchenko EV. Molecular Testing and Targeted Therapy for Non-Small Cell Lung Cancer: Current Status and Perspectives. Crit Rev Oncol Hematol (2021) 157:103194. doi: 10.1016/j.critrevonc.2020.103194 33316418

[2] Bray F Ferlay J Soerjomataram I Siegel RL Torre LA Jemal A. Global Cancer Statistics 2018: GLOBOCAN Estimates of Incidence and Mortality Worldwide for 36 Cancers in 185 Countries. CA Cancer J Clin (2018) 68(6):394–424. doi: 10.3322/caac.21492 30207593

[3] Herbst RS Morgensztern D Boshoff C. The Biology and Management of Non-Small Cell Lung Cancer. Nature (2018) 553(7689):446–54. doi: 10.1038/nature25183 29364287

[4] Molina JR Yang P Cassivi SD Schild SE Adjei AA. Non-Small Cell Lung Cancer: Epidemiology, Risk Factors, Treatment, and Survivorship. Mayo Clin Proc (2008) 83(5):584–94. doi: 10.4065/83.5.584 PMC2718421 18452692

[5] Cancer Genome Atlas Research N. Author Correction: Comprehensive Molecular Profiling of Lung Adenocarcinoma. Nature (2018) 559(7715):E12. doi: 10.1038/s41586-018-0228-6 29925941

[6] Cancer Genome Atlas Research N. Comprehensive Genomic Characterization of Squamous Cell Lung Cancers. Nature (2012) 489(7417):519–25. doi: 10.1038/nature11404 PMC3466113 22960745

[7] Lemmon MA Schlessinger J Ferguson KM. The EGFR Family: Not So Prototypical Receptor Tyrosine Kinases. Cold Spring Harb Perspect Biol (2014) 6(4):a020768. doi: 10.1101/cshperspect.a020768 24691965 PMC3970421

[8] Lin JJ Riely GJ Shaw AT. Targeting ALK: Precision Medicine Takes on Drug Resistance. Cancer Discov (2017) 7(2):137–55. doi: 10.1158/2159-8290.CD-16-1123 PMC5296241 28122866

[9] Shaw AT Ou SH Bang YJ Camidge DR Solomon BJ Salgia R. Crizotinib in ROS1-Rearranged Non-Small-Cell Lung Cancer. N Engl J Med (2014) 371(21):1963–71. doi: 10.1056/NEJMoa1406766 PMC4264527 25264305

[10] Hyman DM Puzanov I Subbiah V Faris JE Chau I Blay JY. Vemurafenib in Multiple Nonmelanoma Cancers With BRAF V600 Mutations. N Engl J Med (2015) 373(8):726–36. doi: 10.1056/NEJMoa1502309 PMC4971773 26287849

[11] Paik PK Drilon A Fan PD Yu H Rekhtman N Ginsberg MS. Response to MET Inhibitors in Patients With Stage IV Lung Adenocarcinomas Harboring MET Mutations Causing Exon 14 Skipping. Cancer Discov (2015) 5(8):842–9. doi: 10.1158/2159-8290.CD-14-1467 PMC4658654 25971939

[12] Leach DR Krummel MF Allison JP. Enhancement of Antitumor Immunity by CTLA-4 Blockade. Science (1996) 271(5256):1734–6. doi: 10.1126/science.271.5256.1734 8596936

[13] McGranahan N Furness AJ Rosenthal R Ramskov S Lyngaa R Saini SK. Clonal Neoantigens Elicit T Cell Immunoreactivity and Sensitivity to Immune Checkpoint Blockade. Science (2016) 351(6280):1463–9. doi: 10.1126/science.aaf1490 PMC4984254 26940869

[14] Amaravadi RK Kimmelman AC Debnath J. Targeting Autophagy in Cancer: Recent Advances and Future Directions. Cancer Discov (2019) 9(9):1167–81. doi: 10.1158/2159-8290.CD-19-0292 PMC7306856 31434711

[15] Levine B Klionsky DJ. Autophagy Wins the 2016 Nobel Prize in Physiology or Medicine: Breakthroughs in Baker's Yeast Fuel Advances in Biomedical Research. Proc Natl Acad Sci USA (2017) 114(2):201–5. doi: 10.1073/pnas.1619876114 PMC5240711 28039434

[16] Bagherniya M Butler AE Barreto GE Sahebkar A. The Effect of Fasting or Calorie Restriction on Autophagy Induction: A Review of the Literature. Ageing Res Rev (2018) 47:183–97. doi: 10.1016/j.arr.2018.08.004 30172870

[17] He L Zhang J Zhao J Ma N Kim SW Qiao S. Autophagy: The Last Defense Against Cellular Nutritional Stress. Adv Nutr (2018) 9(4):493–504. doi: 10.1093/advances/nmy011 30032222 PMC6054220

[18] Mizushima N Levine B Cuervo AM Klionsky DJ. Autophagy Fights Disease Through Cellular Self-Digestion. Nature (2008) 451(7182):1069–75. doi: 10.1038/nature06639 PMC2670399 18305538

[19] Klionsky DJ Abdelmohsen K Abe A Abedin MJ Abeliovich H Acevedo Arozena A. Guidelines for the Use and Interpretation of Assays for Monitoring Autophagy (3rd Edition). Autophagy (2016) 12(1):1–222. doi: 10.1080/15548627.2015.1100356 26799652 PMC4835977

[20] Orenstein SJ Cuervo AM. Chaperone-Mediated Autophagy: Molecular Mechanisms and Physiological Relevance. Semin Cell Dev Biol (2010) 21(7):719–26. doi: 10.1016/j.semcdb.2010.02.005 PMC2914824 20176123

[21] Mukhopadhyay S Mahapatra KK Praharaj PP Patil S Bhutia SK. Recent Progress of Autophagy Signaling in Tumor Microenvironment and Its Targeting for Possible Cancer Therapeutics. Semin Cancer Biol (2021) S1044-579X(21)00227-3. doi: 10.1016/j.semcancer.2021.09.003 34500075

[22] Strohecker AM White E. Targeting Mitochondrial Metabolism by Inhibiting Autophagy in BRAF-Driven Cancers. Cancer Discov (2014) 4(7):766–72. doi: 10.1158/2159-8290.CD-14-0196 PMC4090279 24860158

[23] Yang S Wang X Contino G Liesa M Sahin E Ying H. Pancreatic Cancers Require Autophagy for Tumor Growth. Genes Dev (2011) 25(7):717–29. doi: 10.1101/gad.2016111 PMC3070934 21406549

[24] Yang A Kimmelman AC. Inhibition of Autophagy Attenuates Pancreatic Cancer Growth Independent of TP53/TRP53 Status. Autophagy (2014) 10(9):1683–4. doi: 10.4161/auto.29961 PMC4206544 25046107

[25] Guo JY Teng X Laddha SV Ma S Van Nostrand SC Yang Y. Autophagy Provides Metabolic Substrates to Maintain Energy Charge and Nucleotide Pools in Ras-Driven Lung Cancer Cells. Genes Dev (2016) 30(15):1704–17. doi: 10.1101/gad.283416.116 PMC5002976 27516533

[26] Guo JY Xia B White E. Autophagy-Mediated Tumor Promotion. Cell (2013) 155(6):1216–9. doi: 10.1016/j.cell.2013.11.019 PMC3987898 24315093

[27] Tanida I. Autophagosome Formation and Molecular Mechanism of Autophagy. Antioxid Redox Signal (2011) 14(11):2201–14. doi: 10.1089/ars.2010.3482 20712405

[28] Behrends C Sowa ME Gygi SP Harper JW. Network Organization of the Human Autophagy System. Nature (2010) 466(7302):68–76. doi: 10.1038/nature09204 20562859 PMC2901998

[29] Shibutani ST Yoshimori T. A Current Perspective of Autophagosome Biogenesis. Cell Res (2014) 24(1):58–68. doi: 10.1038/cr.2013.159 24296784 PMC3879706

[30] Ravikumar B Moreau K Jahreiss L Puri C Rubinsztein DC. Plasma Membrane Contributes to the Formation of Pre-Autophagosomal Structures. Nat Cell Biol (2010) 12(8):747–57. doi: 10.1038/ncb2078 PMC2923063 20639872

[31] Hailey DW Rambold AS Satpute-Krishnan P Mitra K Sougrat R Kim PK. Mitochondria Supply Membranes for Autophagosome Biogenesis During Starvation. Cell (2010) 141(4):656–67. doi: 10.1016/j.cell.2010.04.009 PMC3059894 20478256

[32] Walczak M Martens S. Dissecting the Role of the Atg12-Atg5-Atg16 Complex During Autophagosome Formation. Autophagy (2013) 9(3):424–5. doi: 10.4161/auto.22931 PMC3590266 23321721

[33] Onorati AV Dyczynski M Ojha R Amaravadi RK. Targeting Autophagy in Cancer. Cancer (2018) 124(16):3307–18. doi: 10.1002/cncr.31335 PMC6108917 29671878

[34] Wilson MI Dooley HC Tooze SA. WIPI2b and Atg16L1: Setting the Stage for Autophagosome Formation. Biochem Soc Trans (2014) 42(5):1327–34. doi: 10.1042/BST20140177 25233411

[35] Kraya AA Piao S Xu X Zhang G Herlyn M Gimotty P. Identification of Secreted Proteins That Reflect Autophagy Dynamics Within Tumor Cells. Autophagy (2015) 11(1):60–74. doi: 10.4161/15548627.2014.984273 25484078 PMC4502670

[36] Ichimura Y Kirisako T Takao T Satomi Y Shimonishi Y Ishihara N. A Ubiquitin-Like System Mediates Protein Lipidation. Nature (2000) 408(6811):488–92. doi: 10.1038/35044114 11100732

[37] Lamark T Kirkin V Dikic I Johansen T. NBR1 and P62 as Cargo Receptors for Selective Autophagy of Ubiquitinated Targets. Cell Cycle (2009) 8(13):1986–90. doi: 10.4161/cc.8.13.8892 19502794

[38] Gatica D Lahiri V Klionsky DJ. Cargo Recognition and Degradation by Selective Autophagy. Nat Cell Biol (2018) 20(3):233–42. doi: 10.1038/s41556-018-0037-z PMC6028034 29476151

[39] Fass E Shvets E Degani I Hirschberg K Elazar Z. Microtubules Support Production of Starvation-Induced Autophagosomes But Not Their Targeting and Fusion With Lysosomes. J Biol Chem (2006) 281(47):36303–16. doi: 10.1074/jbc.M607031200 16963441

[40] Takats S Pircs K Nagy P Varga A Karpati M Hegedus K. Interaction of the HOPS Complex With Syntaxin 17 Mediates Autophagosome Clearance in Drosophila. Mol Biol Cell (2014) 25(8):1338–54. doi: 10.1091/mbc.E13-08-0449 PMC3982998 24554766

[41] Nakamura S Yoshimori T. New Insights Into Autophagosome-Lysosome Fusion. J Cell Sci (2017) 130(7):1209–16. doi: 10.1242/jcs.196352 28302910

[42] Kimmelman AC White E. Autophagy and Tumor Metabolism. Cell Metab (2017) 25(5):1037–43. doi: 10.1016/j.cmet.2017.04.004 PMC5604466 28467923

[43] Qu X Yu J Bhagat G Furuya N Hibshoosh H Troxel A. Promotion of Tumorigenesis by Heterozygous Disruption of the Beclin 1 Autophagy Gene. J Clin Invest (2003) 112(12):1809–20. doi: 10.1172/JCI20039 PMC297002 14638851

[44] Takamura A Komatsu M Hara T Sakamoto A Kishi C Waguri S. Autophagy-Deficient Mice Develop Multiple Liver Tumors. Genes Dev (2011) 25(8):795–800. doi: 10.1101/gad.2016211 21498569 PMC3078705

[45] Yue Z Jin S Yang C Levine AJ Heintz N. Beclin 1, an Autophagy Gene Essential for Early Embryonic Development, Is A Haploinsufficient Tumor Suppressor. Proc Natl Acad Sci USA (2003) 100(25):15077–82. doi: 10.1073/pnas.2436255100 PMC299911 14657337

[46] Degenhardt K Mathew R Beaudoin B Bray K Anderson D Chen G. Autophagy Promotes Tumor Cell Survival and Restricts Necrosis, Inflammation, and Tumorigenesis. Cancer Cell (2006) 10(1):51–64. doi: 10.1016/j.ccr.2006.06.001 16843265 PMC2857533

[47] Karantza-Wadsworth V Patel S Kravchuk O Chen G Mathew R Jin S. Autophagy Mitigates Metabolic Stress and Genome Damage in Mammary Tumorigenesis. Genes Dev (2007) 21(13):1621–35. doi: 10.1101/gad.1565707 PMC1899472 17606641

[48] Mathew R Kongara S Beaudoin B Karp CM Bray K Degenhardt K. Autophagy Suppresses Tumor Progression by Limiting Chromosomal Instability. Genes Dev (2007) 21(11):1367–81. doi: 10.1101/gad.1545107 PMC1877749 17510285

[49] Rao S Yang H Penninger JM Kroemer G. Autophagy in Non-Small Cell Lung Carcinogenesis: A Positive Regulator of Antitumor Immunosurveillance. Autophagy (2014) 10(3):529–31. doi: 10.4161/auto.27643 PMC4077894 24413089

[50] White E. Deconvoluting the Context-Dependent Role for Autophagy in Cancer. Nat Rev Cancer (2012) 12(6):401–10. doi: 10.1038/nrc3262 PMC3664381 22534666

[51] Guo JY Chen HY Mathew R Fan J Strohecker AM Karsli-Uzunbas G. Activated Ras Requires Autophagy to Maintain Oxidative Metabolism and Tumorigenesis. Genes Dev (2011) 25(5):460–70. doi: 10.1101/gad.2016311 PMC3049287 21317241

[52] Lock R Kenific CM Leidal AM Salas E Debnath J. Autophagy-Dependent Production of Secreted Factors Facilitates Oncogenic RAS-Driven Invasion. Cancer Discov (2014) 4(4):466–79. doi: 10.1158/2159-8290.CD-13-0841 PMC3980002 24513958

[53] Guo JY White E. Autophagy Is Required for Mitochondrial Function, Lipid Metabolism, Growth, and Fate of KRAS(G12D)-Driven Lung Tumors. Autophagy (2013) 9(10):1636–8. doi: 10.4161/auto.26123 PMC5424446 23959381

[54] Strohecker AM Guo JY Karsli-Uzunbas G Price SM Chen GJ Mathew R. Autophagy Sustains Mitochondrial Glutamine Metabolism and Growth of BrafV600E-Driven Lung Tumors. Cancer Discov (2013) 3(11):1272–85. doi: 10.1158/2159-8290.CD-13-0397 PMC3823822 23965987

[55] Guo JY Karsli-Uzunbas G Mathew R Aisner SC Kamphorst JJ Strohecker AM. Autophagy Suppresses Progression of K-Ras-Induced Lung Tumors to Oncocytomas and Maintains Lipid Homeostasis. Genes Dev (2013) 27(13):1447–61. doi: 10.1101/gad.219642.113 PMC3713426 23824538

[56] Karsli-Uzunbas G Guo JY Price S Teng X Laddha SV Khor S. Autophagy Is Required for Glucose Homeostasis and Lung Tumor Maintenance. Cancer Discov (2014) 4(8):914–27. doi: 10.1158/2159-8290.CD-14-0363 PMC4125614 24875857

[57] Bustos SO Antunes F Rangel MC Chammas R. Emerging Autophagy Functions Shape the Tumor Microenvironment and Play a Role in Cancer Progression - Implications for Cancer Therapy. Front Oncol (2020) 10:606436. doi: 10.3389/fonc.2020.606436 33324568 PMC7724038

[58] Wu H Huang S Chen Z Liu W Zhou X Zhang D. Hypoxia-Induced Autophagy Contributes to the Invasion of Salivary Adenoid Cystic Carcinoma Through the HIF-1alpha/BNIP3 Signaling Pathway. Mol Med Rep (2015) 12(5):6467–74. doi: 10.3892/mmr.2015.4255 PMC4626194 26323347

[59] Singla M Bhattacharyya S. Autophagy as a Potential Therapeutic Target During Epithelial to Mesenchymal Transition in Renal Cell Carcinoma: An *In Vitro* Study. BioMed Pharmacother (2017) 94:332–40. doi: 10.1016/j.biopha.2017.07.070 28772211

[60] Bocci F Gearhart-Serna L Boareto M Ribeiro M Ben-Jacob E Devi GR. Toward Understanding Cancer Stem Cell Heterogeneity in the Tumor Microenvironment. Proc Natl Acad Sci USA (2019) 116(1):148–57. doi: 10.1073/pnas.1815345116 PMC6320545 30587589

[61] Wang J Liu D Sun Z Ye T Li J Zeng B. Autophagy Augments the Self-Renewal of Lung Cancer Stem Cells by the Degradation of Ubiquitinated P53. Cell Death Dis (2021) 12(1):98. doi: 10.1038/s41419-021-03392-6 33468994 PMC7815724

[62] Zhou Q Cui F Lei C Ma S Huang J Wang X. ATG7-Mediated Autophagy Involves in miR-138-5p Regulated Self-Renewal and Invasion of Lung Cancer Stem-Like Cells Derived From A549 Cells. Anticancer Drugs (2021) 32(4):376–85. doi: 10.1097/CAD.0000000000000979 33323682

[63] Turley SJ Cremasco V Astarita JL. Immunological Hallmarks of Stromal Cells in the Tumour Microenvironment. Nat Rev Immunol (2015) 15(11):669–82. doi: 10.1038/nri3902 26471778

[64] Kalluri R. The Biology and Function of Fibroblasts in Cancer. Nat Rev Cancer (2016) 16(9):582–98. doi: 10.1038/nrc.2016.73 27550820

[65] Yan Y Chen X Wang X Zhao Z Hu W Zeng S. The Effects and the Mechanisms of Autophagy on the Cancer-Associated Fibroblasts in Cancer. J Exp Clin Cancer Res (2019) 38(1):171. doi: 10.1186/s13046-019-1172-5 31014370 PMC6480893

[66] Rojas-Sanchez G Cotzomi-Ortega I Pazos-Salazar NG Reyes-Leyva J Maycotte P. Autophagy and Its Relationship to Epithelial to Mesenchymal Transition: When Autophagy Inhibition for Cancer Therapy Turns Counterproductive. Biol (Basel) (2019) 8(4):71. doi: 10.3390/biology8040071 PMC6956138 31554173

[67] Marcucci F Ghezzi P Rumio C. The Role of Autophagy in the Cross-Talk Between Epithelial-Mesenchymal Transitioned Tumor Cells and Cancer Stem-Like Cells. Mol Cancer (2017) 16(1):3. doi: 10.1186/s12943-016-0573-8 28137290 PMC5282816

[68] Alizadeh J Glogowska A Thliveris J Kalantari F Shojaei S Hombach-Klonisch S. Autophagy Modulates Transforming Growth Factor Beta 1 Induced Epithelial to Mesenchymal Transition in Non-Small Cell Lung Cancer Cells. Biochim Biophys Acta Mol Cell Res (2018) 1865(5):749–68. doi: 10.1016/j.bbamcr.2018.02.007 29481833

[69] Shen H Yin L Deng G Guo C Han Y Li Y. Knockdown of Beclin-1 Impairs Epithelial-Mesenchymal Transition of Colon Cancer Cells. J Cell Biochem (2018) 119(8):7022–31. doi: 10.1002/jcb.26912 29738069

[70] Peng YF Shi YH Ding ZB Ke AW Gu CY Hui B. Autophagy Inhibition Suppresses Pulmonary Metastasis of HCC in Mice *via* Impairing Anoikis Resistance and Colonization of HCC Cells. Autophagy (2013) 9(12):2056–68. doi: 10.4161/auto.26398 24157892

[71] Sun M Zhao S Duan Y Ma Y Wang Y Ji H. GLUT1 Participates in Tamoxifen Resistance in Breast Cancer Cells Through Autophagy Regulation. Naunyn Schmiedebergs Arch Pharmacol (2021) 394(1):205–16. doi: 10.1007/s00210-020-01893-3 32500187

[72] Roberts DJ Tan-Sah VP Ding EY Smith JM Miyamoto S. Hexokinase-II Positively Regulates Glucose Starvation-Induced Autophagy Through TORC1 Inhibition. Mol Cell (2014) 53(4):521–33. doi: 10.1016/j.molcel.2013.12.019 PMC3943874 24462113

[73] Chen G Zhang Y Liang J Li W Zhu Y Zhang M. Deregulation of Hexokinase II Is Associated With Glycolysis, Autophagy, and the Epithelial-Mesenchymal Transition in Tongue Squamous Cell Carcinoma Under Hypoxia. BioMed Res Int (2018) 2018:8480762. doi: 10.1155/2018/8480762 29682563 PMC5841093

[74] Mukhopadhyay S Biancur DE Parker SJ Yamamoto K Banh RS Paulo JA. Autophagy Is Required for Proper Cysteine Homeostasis in Pancreatic Cancer Through Regulation of SLC7A11. Proc Natl Acad Sci USA (2021) 118(6):e2021475118. doi: 10.1073/pnas.2021475118 33531365 PMC8017731

[75] Chen Y McMillan-Ward E Kong J Israels SJ Gibson SB. Mitochondrial Electron-Transport-Chain Inhibitors of Complexes I and II Induce Autophagic Cell Death Mediated by Reactive Oxygen Species. J Cell Sci (2007) 120(Pt 23):4155–66. doi: 10.1242/jcs.011163 18032788

[76] Scherz-Shouval R Shvets E Fass E Shorer H Gil L Elazar Z. Reactive Oxygen Species Are Essential for Autophagy and Specifically Regulate the Activity of Atg4. EMBO J (2007) 26(7):1749–60. doi: 10.1038/sj.emboj.7601623 PMC1847657 17347651

[77] Dewaele M Maes H Agostinis P. ROS-Mediated Mechanisms of Autophagy Stimulation and Their Relevance in Cancer Therapy. Autophagy (2010) 6(7):838–54. doi: 10.4161/auto.6.7.12113 20505317

[78] Mathew R Karp CM Beaudoin B Vuong N Chen G Chen HY. Autophagy Suppresses Tumorigenesis Through Elimination of P62. Cell (2009) 137(6):1062–75. doi: 10.1016/j.cell.2009.03.048 PMC2802318 19524509

[79] Nihira K Miki Y Iida S Narumi S Ono K Iwabuchi E. An Activation of LC3A-Mediated Autophagy Contributes to *De Novo* and Acquired Resistance to EGFR Tyrosine Kinase Inhibitors in Lung Adenocarcinoma. J Pathol (2014) 234(2):277–88. doi: 10.1002/path.4354 24687913

[80] Li YY Lam SK Mak JC Zheng CY Ho JC. Erlotinib-Induced Autophagy in Epidermal Growth Factor Receptor Mutated Non-Small Cell Lung Cancer. Lung Cancer (2013) 81(3):354–61. doi: 10.1016/j.lungcan.2013.05.012 23769318

[81] Zou Y Ling YH Sironi J Schwartz EL Perez-Soler R Piperdi B. The Autophagy Inhibitor Chloroquine Overcomes the Innate Resistance of Wild-Type EGFR Non-Small-Cell Lung Cancer Cells to Erlotinib. J Thorac Oncol (2013) 8(6):693–702. doi: 10.1097/JTO.0b013e31828c7210 23575415 PMC3855301

[82] Bouhsira E Franc M Lienard E Bouillin C Gandoin C Geurden T. The Efficacy of a Selamectin (Stronghold ®) Spot on Treatment in the Prevention of Bartonella Henselae Transmission by Ctenocephalides Felis in Cats, Using a New High-Challenge Model. Parasitol Res (2015) 114(3):1045–50. doi: 10.1007/s00436-014-4271-4 PMC4336412 25582566

[83] Han W Pan H Chen Y Sun J Wang Y Li J. EGFR Tyrosine Kinase Inhibitors Activate Autophagy as a Cytoprotective Response in Human Lung Cancer Cells. PloS One (2011) 6(6):e18691. doi: 10.1371/journal.pone.0018691 21655094 PMC3107207

[84] Liu JT Li WC Gao S Wang F Li XQ Yu HQ. Autophagy Inhibition Overcomes the Antagonistic Effect Between Gefitinib and Cisplatin in Epidermal Growth Factor Receptor Mutant Non–Small-Cell Lung Cancer Cells. Clin Lung Cancer (2015) 16(5):e55-66. doi: 10.1016/j.cllc.2015.03.006 25979647

[85] Ji C Zhang L Cheng Y Patel R Wu H Zhang Y. Induction of Autophagy Contributes to Crizotinib Resistance in ALK-Positive Lung Cancer. Cancer Biol Ther (2014) 15(5):570–7. doi: 10.4161/cbt.28162 PMC4026079 24556908

[86] Mizushima N Klionsky DJ. Protein Turnover *via* Autophagy: Implications for Metabolism. Annu Rev Nutr (2007) 27:19–40. doi: 10.1146/annurev.nutr.27.061406.093749 17311494

[87] Gorzalczany Y Gilad Y Amihai D Hammel I Sagi-Eisenberg R Merimsky O. Combining an EGFR Directed Tyrosine Kinase Inhibitor With Autophagy-Inducing Drugs: A Beneficial Strategy to Combat Non-Small Cell Lung Cancer. Cancer Lett (2011) 310(2):207–15. doi: 10.1016/j.canlet.2011.07.002 21807458

[88] Amaravadi RK Lippincott-Schwartz J Yin XM Weiss WA Takebe N Timmer W. Principles and Current Strategies for Targeting Autophagy for Cancer Treatment. Clin Cancer Res (2011) 17(4):654–66. doi: 10.1158/1078-0432.CCR-10-2634 PMC3075808 21325294

[89] Yamamoto K Venida A Yano J Biancur DE Kakiuchi M Gupta S. Autophagy Promotes Immune Evasion of Pancreatic Cancer by Degrading MHC-I. Nature (2020) 581(7806):100–5. doi: 10.1038/s41586-020-2229-5 PMC7296553 32376951

[90] Yamamoto K Venida A Perera RM Kimmelman AC. Selective Autophagy of MHC-I Promotes Immune Evasion of Pancreatic Cancer. Autophagy (2020) 16(8):1524–5. doi: 10.1080/15548627.2020.1769973 PMC7469632 32459143

[91] Noman MZ Parpal S Van Moer K Xiao M Yu Y Viklund J. Inhibition of Vps34 Reprograms Cold Into Hot Inflamed Tumors and Improves Anti-PD-1/PD-L1 Immunotherapy. Sci Adv (2020) 6(18):eaax7881. doi: 10.1126/sciadv.aax7881 32494661 PMC7190323

[92] Baginska J Viry E Berchem G Poli A Noman MZ van Moer K. Granzyme B Degradation by Autophagy Decreases Tumor Cell Susceptibility to Natural Killer-Mediated Lysis Under Hypoxia. Proc Natl Acad Sci USA (2013) 110(43):17450–5. doi: 10.1073/pnas.1304790110 PMC3808626 24101526

[93] Noman MZ Janji B Kaminska B Van Moer K Pierson S Przanowski P. Blocking Hypoxia-Induced Autophagy in Tumors Restores Cytotoxic T-Cell Activity and Promotes Regression. Cancer Res (2011) 71(18):5976–86. doi: 10.1158/0008-5472.Can-11-1094 21810913

[94] Zamame Ramirez JA Romagnoli GG Falasco BF Gorgulho CM Sanzochi Fogolin C Dos Santos DC. Blocking Drug-Induced Autophagy With Chloroquine in HCT-116 Colon Cancer Cells Enhances DC Maturation and T Cell Responses Induced by Tumor Cell Lysate. Int Immunopharmacol (2020) 84:106495. doi: 10.1016/j.intimp.2020.106495 32298965 PMC7152898

[95] Guerriero JL. Macrophages: Their Untold Story in T Cell Activation and Function. Int Rev Cell Mol Biol (2019) 342:73–93. doi: 10.1016/bs.ircmb.2018.07.001 30635094

[96] Ma F Ding MG Lei YY Luo LH Jiang S Feng YH. SKIL Facilitates Tumorigenesis and Immune Escape of NSCLC *via* Upregulating TAZ/autophagy Axis. Cell Death Dis (2020) 11(12):1028. doi: 10.1038/s41419-020-03200-7 33268765 PMC7710697

[97] Wei J Long L Yang K Guy C Shrestha S Chen Z. Autophagy Enforces Functional Integrity of Regulatory T Cells by Coupling Environmental Cues and Metabolic Homeostasis. Nat Immunol (2016) 17(3):277–85. doi: 10.1038/ni.3365 PMC4755832 26808230

[98] Parker KH Horn LA Ostrand-Rosenberg S. High-Mobility Group Box Protein 1 Promotes the Survival of Myeloid-Derived Suppressor Cells by Inducing Autophagy. J Leukoc Biol (2016) 100(3):463–70. doi: 10.1189/jlb.3HI0715-305R PMC4982609 26864266

[99] Li W Tanikawa T Kryczek I Xia H Li G Wu K. Aerobic Glycolysis Controls Myeloid-Derived Suppressor Cells and Tumor Immunity *via* a Specific CEBPB Isoform in Triple-Negative Breast Cancer. Cell Metab (2018) 28(1):87–103.e6. doi: 10.1016/j.cmet.2018.04.022 29805099 PMC6238219

[100] Chen D Xie J Fiskesund R Dong W Liang X Lv J. Chloroquine Modulates Antitumor Immune Response by Resetting Tumor-Associated Macrophages Toward M1 Phenotype. Nat Commun (2018) 9(1):873. doi: 10.1038/s41467-018-03225-9 29491374 PMC5830447

[101] Cunha LD Yang M Carter R Guy C Harris L Crawford JC. LC3-Associated Phagocytosis in Myeloid Cells Promotes Tumor Immune Tolerance. Cell (2018) 175(2):429–441.e16. doi: 10.1016/j.cell.2018.08.061 30245008 PMC6201245

[102] Schmitz KJ Ademi C Bertram S Schmid KW Baba HA. Prognostic Relevance of Autophagy-Related Markers LC3, P62/Sequestosome 1, Beclin-1 and ULK1 in Colorectal Cancer Patients With Respect to KRAS Mutational Status. World J Surg Oncol (2016) 14(1):189–9. doi: 10.1186/s12957-016-0946-x PMC4957418 27444698

[103] Lazova R Camp RL Klump V Siddiqui SF Amaravadi RK Pawelek JM. Punctate LC3B Expression Is A Common Feature of Solid Tumors and Associated With Proliferation, Metastasis, and Poor Outcome. Clin Cancer Res (2012) 18(2):370–9. doi: 10.1158/1078-0432.Ccr-11-1282 PMC4825867 22080440

[104] Wu DH Jia CC Chen J Lin ZX Ruan DY Li X. Autophagic LC3B Overexpression Correlates With Malignant Progression and Predicts a Poor Prognosis in Hepatocellular Carcinoma. Tumour Biol (2014) 35(12):12225–33. doi: 10.1007/s13277-014-2531-7 25256671

[105] Schläfli AM Adams O Galván JA Gugger M Savic S Bubendorf L. Prognostic Value of the Autophagy Markers LC3 and P62/SQSTM1 in Early-Stage Non-Small Cell Lung Cancer. Oncotarget (2016) 7(26):39544–55. doi: 10.18632/oncotarget.9647 PMC5129952 27250032

[106] Sivridis E Koukourakis MI Zois CE Ledaki I Ferguson DJ Harris AL. LC3A-Positive Light Microscopy Detected Patterns of Autophagy and Prognosis in Operable Breast Carcinomas. Am J Pathol (2010) 176(5):2477–89. doi: 10.2353/ajpath.2010.090049 PMC2861112 20382705

[107] Karpathiou G Sivridis E Koukourakis MI Mikroulis D Bouros D Froudarakis ME. Light-Chain 3A Autophagic Activity and Prognostic Significance in Non-Small Cell Lung Carcinomas. Chest (2011) 140(1):127–34. doi: 10.1378/chest.10-1831 21148243

[108] Wang X Du Z Li L Shi M Yu Y. Beclin 1 and P62 Expression in Non-Small Cell Lung Cancer: Relation With Malignant Behaviors and Clinical Outcome. Int J Clin Exp Pathol (2015) 8(9):10644–52. PMC4637589 26617774

[109] Inoue D Suzuki T Mitsuishi Y Miki Y Suzuki S Sugawara S. Accumulation of P62/SQSTM1 Is Associated With Poor Prognosis in Patients With Lung Adenocarcinoma. Cancer Sci (2012) 103(4):760–6. doi: 10.1111/j.1349-7006.2012.02216.x PMC7659245 22320446

[110] Wu DH Wang TT Ruan DY Li X Chen ZH Wen JY. Combination of ULK1 and LC3B Improve Prognosis Assessment of Hepatocellular Carcinoma. BioMed Pharmacother (2018) 97:195–202. doi: 10.1016/j.biopha.2017.10.025 29091866

[111] Zhou W Yue C Deng J Hu R Xu J Feng L. Autophagic Protein Beclin 1 Serves as an Independent Positive Prognostic Biomarker for Non-Small Cell Lung Cancer. PloS One (2013) 8(11):e80338. doi: 10.1371/journal.pone.0080338 24260370 PMC3829868

[112] Zheng T Li D He Z Feng S Zhao S. Prognostic and Clinicopathological Significance of Beclin-1 in Non-Small-Cell Lung Cancer: A Meta-Analysis. Onco Targets Ther (2018) 11:4167–75. doi: 10.2147/ott.S164987 PMC6056151 30050308

[113] Liu PF Chen HC Cheng JS Tsai WL Lee HP Wang SC. Association of ATG4B and Phosphorylated ATG4B Proteins With Tumorigenesis and Prognosis in Oral Squamous Cell Carcinoma. Cancers (Basel) (2019) 11(12):1854. doi: 10.3390/cancers11121854 PMC6966594 31771238

[114] Losmanová T Janser FA Humbert M Tokarchuk I Schläfli AM Neppl C. Chaperone-Mediated Autophagy Markers LAMP2A and HSC70 Are Independent Adverse Prognostic Markers in Primary Resected Squamous Cell Carcinomas of the Lung. Oxid Med Cell Longev (2020) 2020:8506572. doi: 10.1155/2020/8506572 33029283 PMC7527932

[115] Jiang A Liu N Bai S Wang J Gao H Zheng X. Identification and Validation of an Autophagy-Related Long Non-Coding RNA Signature as a Prognostic Biomarker for Patients With Lung Adenocarcinoma. J Thorac Dis (2021) 13(2):720–34. doi: 10.21037/jtd-20-2803 PMC7947511 33717544

[116] Puissant A Fenouille N Auberger P. When Autophagy Meets Cancer Through P62/SQSTM1. Am J Cancer Res (2012) 2(4):397–413. 22860231 PMC3410580

[117] Lin MG Hurley JH. Structure and Function of the ULK1 Complex in Autophagy. Curr Opin Cell Biol (2016) 39:61–8. doi: 10.1016/j.ceb.2016.02.010 PMC4828305 26921696

[118] Egan DF Chun MG Vamos M Zou H Rong J Miller CJ. Small Molecule Inhibition of the Autophagy Kinase ULK1 and Identification of ULK1 Substrates. Mol Cell (2015) 59(2):285–97. doi: 10.1016/j.molcel.2015.05.031 PMC4530630 26118643

[119] Petherick KJ Conway OJ Mpamhanga C Osborne SA Kamal A Saxty B. Pharmacological Inhibition of ULK1 Kinase Blocks Mammalian Target of Rapamycin (mTOR)-Dependent Autophagy. J Biol Chem (2015) 290(18):11376–83. doi: 10.1074/jbc.C114.627778 PMC4416842 25833948

[120] Nguyen TG Honson NS Arns S Davis TL Dhe-Paganon S Kovacic S. Development of Fluorescent Substrates and Assays for the Key Autophagy-Related Cysteine Protease Enzyme, ATG4B. Assay Drug Dev Technol (2014) 12(3):176–89. doi: 10.1089/adt.2013.561 PMC3994995 24735444

[121] Akin D Wang SK Habibzadegah-Tari P Law B Ostrov D Li M. A Novel ATG4B Antagonist Inhibits Autophagy and has a Negative Impact on Osteosarcoma Tumors. Autophagy (2014) 10(11):2021–35. doi: 10.4161/auto.32229 PMC4502682 25483883

[122] Wu S Su J Qian H Guo T. SLC27A4 Regulate ATG4B Activity and Control Reactions to Chemotherapeutics-Induced Autophagy in Human Lung Cancer Cells. Tumour Biol (2016) 37(5):6943–52. doi: 10.1007/s13277-015-4587-4 26662804

[123] Mizushima N Yoshimori T Ohsumi Y. The Role of Atg Proteins in Autophagosome Formation. Annu Rev Cell Dev Biol (2011) 27:107–32. doi: 10.1146/annurev-cellbio-092910-154005 21801009

[124] Levine B Sinha S Kroemer G. Bcl-2 Family Members: Dual Regulators of Apoptosis and Autophagy. Autophagy (2008) 4(5):600–6. doi: 10.4161/auto.6260 PMC2749577 18497563

[125] Moon EK Kim SH Hong Y Chung DI Goo YK Kong HH. Autophagy Inhibitors as a Potential Antiamoebic Treatment for Acanthamoeba Keratitis. Antimicrob Agents Chemother (2015) 59(7):4020–5. doi: 10.1128/aac.05165-14 PMC4468686 25896709

[126] Mao X Nanzhang Xiao J Wu H Ding K. Hypoxia-Induced Autophagy Enhances Cisplatin Resistance in Human Bladder Cancer Cells by Targeting Hypoxia-Inducible Factor-1α. J Immunol Res (2021) 2021:8887437. doi: 10.1155/2021/8887437 33681390 PMC7904373

[127] McAfee Q Zhang Z Samanta A Levi SM Ma X-H Piao S. Autophagy Inhibitor Lys05 has Single-Agent Antitumor Activity and Reproduces the Phenotype of a Genetic Autophagy Deficiency. Proc Natl Acad Sci USA (2012) 109(21):8253–8. doi: 10.1073/pnas.1118193109 PMC3361415 22566612

[128] Goodall ML Wang T Martin KR Kortus MG Kauffman AL Trent JM. Development of Potent Autophagy Inhibitors That Sensitize Oncogenic BRAF V600E Mutant Melanoma Tumor Cells to Vemurafenib. Autophagy (2014) 10(6):1120–36. doi: 10.4161/auto.28594 PMC4091172 24879157

[129] Pellegrini P Strambi A Zipoli C Hägg-Olofsson M Buoncervello M Linder S. Acidic Extracellular pH Neutralizes the Autophagy-Inhibiting Activity of Chloroquine: Implications for Cancer Therapies. Autophagy (2014) 10(4):562–71. doi: 10.4161/auto.27901 PMC3984580 24492472

[130] Wang T Goodall ML Gonzales P Sepulveda M Martin KR Gately S. Synthesis of Improved Lysomotropic Autophagy Inhibitors. J Med Chem (2015) 58(7):3025–35. doi: 10.1021/jm501586m 25793774

[131] Mauvezin C Neufeld TP. Bafilomycin A1 Disrupts Autophagic Flux by Inhibiting Both V-ATPase-Dependent Acidification and Ca-P60A/SERCA-Dependent Autophagosome-Lysosome Fusion. Autophagy (2015) 11(8):1437–8. doi: 10.1080/15548627.2015.1066957 PMC4590655 26156798

[132] Yang YP Hu LF Zheng HF Mao CJ Hu WD Xiong KP. Application and Interpretation of Current Autophagy Inhibitors and Activators. Acta Pharmacol Sin (2013) 34(5):625–35. doi: 10.1038/aps.2013.5 PMC4002883 23524572

[133] Briceño E Reyes S Sotelo J. Therapy of Glioblastoma Multiforme Improved by the Antimutagenic Chloroquine. Neurosurg Focus (2003) 14(2):e3. doi: 10.3171/foc.2003.14.2.4 15727424

[134] Sotelo J Briceño E López-González MA. Adding Chloroquine to Conventional Treatment for Glioblastoma Multiforme: A Randomized, Double-Blind, Placebo-Controlled Trial. Ann Intern Med (2006) 144(5):337–43. doi: 10.7326/0003-4819-144-5-200603070-00008 16520474

[135] Goldberg SB Supko JG Neal JW Muzikansky A Digumarthy S Fidias P. A Phase I Study of Erlotinib and Hydroxychloroquine in Advanced Non-Small-Cell Lung Cancer. J Thorac Oncol (2012) 7(10):1602–8. doi: 10.1097/JTO.0b013e318262de4a PMC3791327 22878749

[136] Rojas-Puentes LL Gonzalez-Pinedo M Crismatt A Ortega-Gomez A Gamboa-Vignolle C Nuñez-Gomez R. Phase II Randomized, Double-Blind, Placebo-Controlled Study of Whole-Brain Irradiation With Concomitant Chloroquine for Brain Metastases. Radiat Oncol (2013) 8:209. doi: 10.1186/1748-717x-8-209 24010771 PMC3848663

[137] Malhotra J Jabbour S Orlick M Riedlinger G Guo Y White E. Phase Ib/II Study of Hydroxychloroquine in Combination With Chemotherapy in Patients With Metastatic Non-Small Cell Lung Cancer (NSCLC). Cancer Treat Res Commun (2019) 21:100158. doi: 10.1016/j.ctarc.2019.100158 31521049

[138] Pasquier B. Autophagy Inhibitors. Cell Mol Life Sci (2016) 73(5):985–1001. doi: 10.1007/s00018-015-2104-y 26658914 PMC11108294

[139] Guo JY White E. Autophagy, Metabolism, and Cancer. Cold Spring Harb Symp Quant Biol (2016) 81:73–8. doi: 10.1101/sqb.2016.81.030981 PMC5521269 28209717

